# De novo identification of viral pathogens from cell culture hologenomes

**DOI:** 10.1186/1756-0500-5-11

**Published:** 2012-01-06

**Authors:** Ashok Patowary, Rajendra Kumar Chauhan, Meghna Singh, Shamsudheen KV, Vinita Periwal, Kushwaha KP, Gajanand N Sapkal, Vijay P Bondre, Milind M Gore, Sridhar Sivasubbu, Vinod Scaria

**Affiliations:** 1 CSIR Institute of Genomics and Integrative Biology (CSIR-IGIB), Mall Road, Delhi 110007, India; 2 BRD Medical College and Nehru Hospital, Gorakhpur, Uttar Pradesh, India; 3 National Institute of Virology (ICMR), Pune, India; 4 National Institute of Virology (ICMR), Gorakhpur Unit, Gorakhpur, India

**Keywords:** Epidemics, Mixed Population Genomes, Hologenome, De novo assembly, Japanese encephalitis, Next generation sequencing

## Abstract

**Background:**

Fast, specific identification and surveillance of pathogens is the cornerstone of any outbreak response system, especially in the case of emerging infectious diseases and viral epidemics. This process is generally tedious and time-consuming thus making it ineffective in traditional settings. The added complexity in these situations is the non-availability of pure isolates of pathogens as they are present as mixed genomes or hologenomes. Next-generation sequencing approaches offer an attractive solution in this scenario as it provides adequate depth of sequencing at fast and affordable costs, apart from making it possible to decipher complex interactions between genomes at a scale that was not possible before. The widespread application of next-generation sequencing in this field has been limited by the non-availability of an efficient computational pipeline to systematically analyze data to delineate pathogen genomes from mixed population of genomes or hologenomes.

**Findings:**

We applied next-generation sequencing on a sample containing mixed population of genomes from an epidemic with appropriate processing and enrichment. The data was analyzed using an extensive computational pipeline involving mapping to reference genome sets and *de-novo *assembly. In depth analysis of the data generated revealed the presence of sequences corresponding to *Japanese encephalitis *virus. The genome of the virus was also independently *de-novo *assembled. The presence of the virus was in addition, verified using standard molecular biology techniques.

**Conclusions:**

Our approach can accurately identify causative pathogens from cell culture hologenome samples containing mixed population of genomes and in principle can be applied to patient hologenome samples without any background information. This methodology could be widely applied to identify and isolate pathogen genomes and understand their genomic variability during outbreaks.

## Findings

Viral pathogens have been a major cause of epidemics worldwide [1-3]. The regular surveillance system in many cases of viral epidemics is ineffective to directly identify the virus unless directed by telltale clinical features and clinical complications [4,5]. In many cases the disease causing pathogen is not identified [6], and contributes to the inadequate and inappropriate management of the condition. Many of the disease outbreaks are also caused by changes in the environmental niche [7], and at least in some cases cause the emergence of new pathogens [6,8-11]. It is estimated that at least 33 new pathogens have emerged during the last three decades [12]. Identification of causative organisms during viral outbreak is a problem, primarily due to the tedious methods involving isolation and culturing of the pathogen [13,14]. The relatively low concentration of the genetic material, especially in the case of RNA viruses and the heavy interference due to the host cell nucleic acid and other metagenomes makes sequencing based approaches ineffective, unless done at higher depths [15,16].

The availability of next-generation sequencing (NGS) technology has enabled the scale and ease of addressing biological questions on a genomics perspective [17,18]. The throughput of sequencing enables deep sequencing of nucleic acids, adequate to provide for enough reads of the pathogen, even while the interference of the host genetic material is very high. Metagenomics has been one of the major applications of NGS technology for understanding the composition and dynamics of mixed population of organisms [19]. The field has now emerged to a vibrant area of genomics trying to understand a large spectrum of environmental niches right from human body in both disease as well as healthy states to natural geographical niches [20,21]. Although NGS technology has been successfully used for addressing a large diversity of biological questions, its application to address questions pertaining to bio-surveillance and emerging infectious diseases on a large scale has been limited [22], in spite of the unprecedented opportunity provided by the scale and speed of operations [17].

Hologenome, a term borrowed from evolutionary biology is defined as the sum of the genetic information of the host and its microbiota [23]. Hologenomics is an emerging field in genomics which deals with mixed population of genomes, as in the case of interacting populations in host-pathogen and commensals. Hologenome differs from the widely popular term Metagenome, which involves the study of communities of microbes directly in their natural environments [24].

Cultured population of viruses co-exists and interacts intricately with their host genomes [23]. Although this presents a technical challenge in isolating individual genomes from mixed populations, it offers enormous possibility to understand the interactions and dynamics between the genomes in real-time.

Here we report the sequencing and analysis methodology, involving computational algorithms for reference mapping and *de novo *sequence assembly to accurately identify viral pathogens from mixed populations of genomes. The pipeline relies on the specificity of sequence mappings and the differential distribution of the mapped reads across genomes. As a proof of concept, we applied the methodology on a cell culture hologenome consisting of human, bacterial and viral genomes, and could specifically identify the viral pathogen. This methodology could potentially be applied for rapid and specific identification of viral pathogens during epidemic outbreaks.

### Sample collection and RNA isolation

The sample was collected and isolated during an epidemic of acute viral encephalitis from an anonymous patient suffering from fever and acute encephalitis from Baba Raghav Das (BRD) Medical College and Nehru Hospital, Gorakhpur, India. Sample was procured and processed as per ethical procedures laid down by BRD Medical College, Gorakhpur, India and National Institute of Virology, Pune, India. Using standard virus isolation protocols samples were inoculated in human Rhabdosarcoma (RD) and Baby Hamster kidney (BHK) cell lines for virus isolation [25,26]. Cells were observed for cytopathological effects (CPE) and passaged three times. The cell culture supernatant was filtered using 0.22 μm Millipore filters for every passage. RNA was isolated using Qiagen (*QiaAMP *viral RNA minikit) kit as per manufacturer's instructions. The RNA was eluted in 60 μlitre of AVE buffer.

### Library preparation, sequencing and genome assembly

The RNA library was prepared according to the manufacturer's instructions using RNA Sample-prep kit (Illumina Inc, USA) for sequencing on Illumina sequencing platform. Two microgram of total RNA was fragmented using divalent cation. Cleaved RNA was converted to cDNA using reverse transcriptase (SSRT-II Invitrogen) and random primers. The fragments were further subjected to second strand cDNA synthesis using DNA polymerase as per manufacturer's instructions. End-repairing process followed by A-base addition and adapter ligation was further performed on the cDNA fragments. Approximately 350 base pair products were separated by gel excision and enriched with PCR to create the final library.

Clusters were generated on the flow cell using cBot Paired end cluster generation kit (Illumina Inc, USA) as per manufacturer's instructions. The sequencing runs were performed on Illumina Inc, USA) using 76 × 2 base reads. The sequence-quality files generated was transformed to Sanger quality scores using custom scripts.

**Figure 1:**
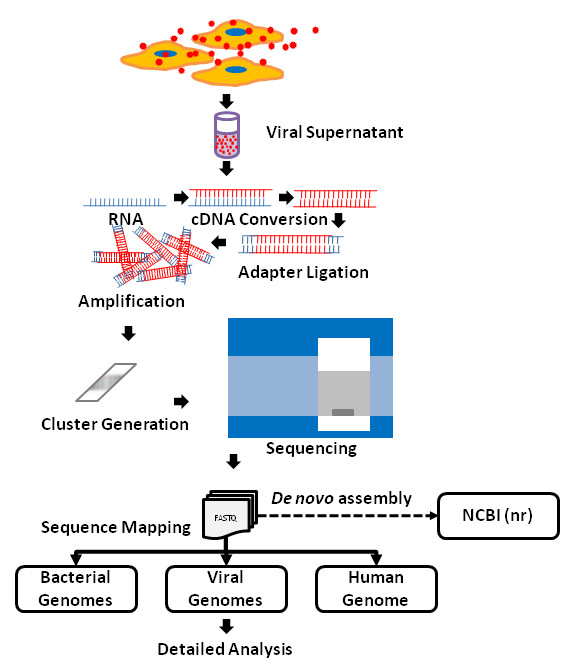
**Workflow**. Summary of the Methodology for viral sample processing and computational analysis.

The paired end reads were mapped to the reference datasets using Mapping and Assembly with Qualities (MAQ) software [27]. The datasets of 3735 viral genomes, 2352 Bacterial genomes and the human genome corresponding to GRch37/hg19 build was downloaded from NCBI [28] and used for mapping. The mapped reads were further analyzed and compared for reads that overlapped in each reference set. The genomes that mapped the maximum number of reads post-alignment were parsed using custom scripts and were further considered for analysis. Single nucleotide variations and Insertion Deletion (InDel) events were called using MAQ scripts. Mappings and functional analysis of the variations were performed using custom scripts. The entire pipeline for the data generation and analysis is summarized in Figure 1.

Velvet [29], a popularly used *de novo *assembly algorithm based on de Bruijn graphs was used for the *de novo *assembly. The entire read data was partitioned into smaller subsets for analysis. *De-novo *assembly was attempted on the subsets with different k-mers. The data was compiled and compared using in-house scripts.

### RT-PCR validation

Experimental validation of the virus was performed using reverse transcription (RT)-polymerase chain reaction. Specific primer JEV PM1R (reverse primer): 5'-CGGARTCTCCTGCTTCGCTTGG-3' and JEV C1F (forward primer): 5'-GGCAGAAAGCAAAACAAAAGA-3' specific for *Japanese encephalitis *virus were used. RNA was initially reverse transcribed using JEV PM1R at 50°C using Superscript II reverse transcriptase (Invitrogen, Life Science Technologies). The cDNA was amplified using forward primer JEV C1F and reverse primer JEV PM1R. PCR amplification was carried out by denaturing 94°C for 5 min, followed by 35 cycles of 94°C for 30 sec 58°C for 30 sec, 72°C for 1 min and final extension of 3 min using *Taq *DNA polymerase (Fermentas).

## Results and discussion

**Figure 2:**
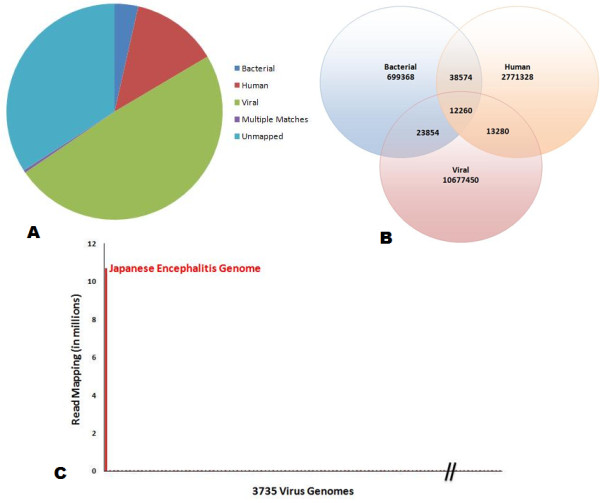
**Reads mapping and analysis**. Mapping and analysis of reads mapping to reference sequence datasets. (**A**) The distribution of reads mapping to each reference dataset. (**B**) Reads mapping to each of the three reference datasets and the overlaps between them. (**C**) Distribution of reads mapping to the viral reference datasets. The horizontal axis depicts the 3735 viral genomes while the vertical axis depicts the reads mapping to each of the viral genomes. The reads that map to *Japanese encephalitis *virus was ordered different from the reads that mapped to the next highest.

We generated approximately 22 million sequence reads from the cell culture hologenome sample [SRA: SRX099040]. All reads were 76 bases in paired end mode, and amounted to a total of 1.67 Gigabases. The paired-end reads had an average insert size of 350 bases. The read statistics is available as Additional file 1. The reads were mapped to Human, 2352 bacteria and 3735 viral genome datasets using Mapping and Assembly with Qualities (MAQ) with default parameters. Maximum of two bases mismatching was permitted in the seed, and the alignments were performed in paired end mode. All the three alignment files were later transformed and the read headers, which overlap in each of the sets, were compared using in-house scripts. We found 2,835,442 reads mapping to the Human genome, comprising of 13% of the entire reads, 774,056 reads comprising of 3.6% of the data mapping to the bacterial reference set, while 10,726,844 mapped to Virus genomes, comprising 49% of the reads (Figure 2a &2b). We also compared the reads that overlapped in each of the reference alignment sets. The overlaps were minimal suggesting specificity of sequence alignments. The reads mapping to the viral sequence dataset comprising of 3735 complete viral reference genomes were further analyzed in depth. The analysis revealed that 99% of the reads mapped to the *Japanese encephalitis *Virus genome (Figure 2c). This comprised of over 10 million reads and provided an effective coverage of over 70,000X over the *Japanese encephalitis *genome.

Reference mapping is an attractive strategy, as it is fast and frugal on compute and memory requirements, however is limited by the availability of the genome under consideration in the reference dataset. This could limit the widespread application of this methodology in identifying pathogens that have not been sequenced before, as in the case of new and emerging infectious diseases. This limitation could be potentially overcome using a *de novo *assembly strategy, which does not rely on prior knowledge of the genome sequence. We therefore attempted *de novo *assembly of the cell culture hologenome using Velvet. Different k-mer values and coverage cut-offs were used for the assembly. The largest contig was assembled at a k-mer of 27 and had a length of 10,758 bases [GenBank: JN644310]. The *de novo *assembled contig aligned with 99% identity to the *Japanese encephalitis *virus isolate 014178 genome in NCBI nr database, having a length of 10,976 bases, further confirming the identity of the virus. We also attempted to validate the identity of virus using specific reverse transcriptase PCR (RT-PCR). JE specific primers mapping in the C-prM region amplified approximately 400 bp amplicon as expected [25,26], substantiating the identity of the virus. The data is represented in Additional file 2.

Furthermore the reads and alignments in the reference assembly that mapped to *Japanese encephalitis *genome were analyzed in depth to understand the genome organization and genomic variations of the isolate. We identified a total of 209 genomic variations in the genome with high confidence. The complete list of variations, their genomic loci, their type (i.e. synonymous and non-synonymous) and the genes that harbor them are summarized in Additional file 3. Fast and specific identification of viral pathogens would enable adequate and timely response to disease outbreaks. Next generation sequencing technology coupled with efficient computational algorithms could be effectively used for the identification of pathogens in such epidemics.

## Conclusions

Here we have used next-generation sequencing approach and generated over 22 million sequence reads from a cell culture hologenome sample consisting of human, bacteria and virus. We also successfully applied a pipeline of computational algorithms including reference mapping and *de novo *assembly to identify the pathogen as *Japanese encephalitis *virus. Furthermore we also validated the identity of the virus using RT-PCR techniques. In summary we successfully demonstrate the utility of such approaches in identification of viral pathogens from mixed population of genomes. As cheaper and faster sequencing technology becomes widely available in research labs, this approach would open up immense opportunities in identifying viral pathogens in outbreaks, apart from being used in regular bio-surveillance of infectious diseases and screening for agents used in bioterrorism. This methodology could also find application in special situations like food processing, beverage, and water quality monitoring for identification of food borne and waterborne pathogens respectively.

## Availability of supporting data

Raw sequence data is available at the NCBI Short Read Archive with ID: SRX099040. Sequence generated using de novo genome assembly tools is available at Genbank with ID: JN644310.

## Competing interests

The authors declare that they have no competing interests.

## Authors' contributions

KKP, GNS, VPB & MMG-Collected the samples; GNS, VPB & MMG-Isolated and inoculated the virus and maintained the cell culture; AP, RK, MS, SKV & SSB-Generated the nucleic acid sequences and conducted the molecular studies; AP, VP & VS-Developed the algorithms and conducted the computational analysis; MMG, SSB & VS-Designed the study, analyzed the results and drafted the manuscript.

## Supplementary Material

Additional file 1 **Read Statistics**. Excel file containing detailed information on various read statistics. Click here for file

Additional file 2 **RT-PCR validations**. JPG image depicting reverse transcriptase validation for *Japanese encephalitis*. Click here for file

Additional file 3 **List of Variations**. Microsoft DOC file containing table on variations identified in Japanese Encephalitis genome (NC_001437). Click here for file
