# Supplementary figures and images for: De novo identification of viral pathogens from cell culture hologenomes
Source: BMC Res Notes. 2012 Jan 6;5:11. doi: 10.1186/1756-0500-5-11 (PMC3284880; doi:10.1186/1756-0500-5-11)

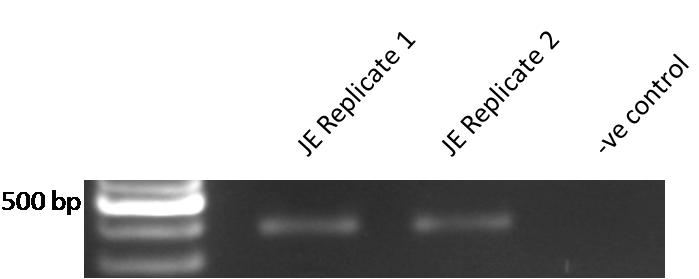

Supplement: Additional file 2 — RT-PCR validations. JPG image depicting reverse transcriptase validation for Japanese encephalitis. [file 1756-0500-5-11-S2.JPEG]
