# Supplementary material for: De novo identification of viral pathogens from cell culture hologenomes
Source: BMC Res Notes. 2012 Jan 6;5:11. doi: 10.1186/1756-0500-5-11 (PMC3284880; doi:10.1186/1756-0500-5-11)
Supplement: Additional file 3 — List of Variations. Microsoft DOC file containing table on variations identified in Japanese Encephalitis genome (NC_001437). [file 1756-0500-5-11-S3.DOC]

**Additional Table:** Variations identified in Japanese Encephalitis genome (NC_001437)

| **Peptide_Id** | **Genomic location** | **Length of peptide** | **Distribution of variations (209)** | |
| --- | --- | --- | --- | --- |
|  |  |  | **Synonymous (191)** | **Non-synonymous (15)** |
| NP_775663 | 96..476 | 127 | 5 | 2 |
| NP_775662 | 96..410 | 105 | 4 | 1 |
| NP_775664 | 477..977 | 167 | 9 | 1 |
| NP_775665 | 753..977 | 75 | 2 | 1 |
| NP_775666 | 978..2477 | 500 | 25 | 1 |
| NP_775667 | 2478..3533 | 352 | 17 | 0 |
| NP_775668 | 3534..4214 | 227 | 13 | 1 |
| NP_775669 | 4215..4607 | 131 | 7 | 0 |
| NP_775670 | 4608..6464 | 619 | 30 | 3 |
| NP_775671 | 6465..6842 | 126 | 6 | 1 |
| NP_775672 | 6843..6911 | 23 | 2 | 0 |
| NP_775673 | 6912..7676 | 255 | 22 | 2 |
| NP_775674 | 7677..10391 | 905 | 49 | 2 |
